# Frontal-Subcortical Volumetric Deficits in Single Episode, Medication-Naïve Depressed Patients and the Effects of 8 Weeks Fluoxetine Treatment: A VBM-DARTEL Study

**DOI:** 10.1371/journal.pone.0079055

**Published:** 2014-01-10

**Authors:** Lingtao Kong, Feng Wu, Yanqing Tang, Ling Ren, Dongyan Kong, Ying Liu, Ke Xu, Fei Wang

**Affiliations:** 1 Department of Psychiatry, The First Affiliated Hospital, China Medical University, Shenyang, Liaoning, PR China; 2 Department of Radiology, The First Affiliated Hospital, China Medical University, Shenyang, Liaoning, PR China; 3 Department of Psychology, Quanzhou First Hospital, Quanzhou, Fujian, PR China; 4 Department of Psychiatry, Yale University School of Medicine, New Haven, Connecticut, United States of America; Beijing Normal University, China

## Abstract

**Background:**

Convergent studies suggest that morphological abnormalities of frontal-subcortical circuits which involved with emotional and cognitive processing may contribute to the pathophysiology of major depressive disorder (MDD). Antidepressant treatment which has been reported to reverse the functional abnormalities of frontal-subcortical circuits in MDD may have treating effects to related brain morphological abnormalities. In this study, we used voxel-based morphometry method to investigate whole brain structural abnormalities in single episode, medication-naïve MDD patients. Furthermore, we investigated the effects of an 8 weeks pharmacotherapy with fluoxetine.

**Methods:**

28 single episode, medication-naïve MDD participants and 28 healthy controls (HC) acquired the baseline high-resolution structural magnetic resonance imaging (sMRI) scan. 24 MDD participants acquired a follow-up sMRI scan after 8 weeks antidepressant treatment. Gray matter volumetric (GMV) difference between groups was examined.

**Results:**

Medication-naïve MDD had significantly decreased GMV in the right dorsolateral prefrontal cortex and left middle frontal gyrus as well as increased GMV in the left thalamus and right insula compared to HC (P<0.05, corrected). Moreover, treated MDD had significantly increased GMV in the left middle frontal gyrus and right orbitofrontal cortex compared to HC (P<0.05, corrected). No difference on GMV was detected between medication-naïve MDD group and treated MDD group.

**Conclusions:**

This study of single episode, medication-naïve MDD subjects demonstrated structural abnormalities of frontal-subcortical circuitsin the early stage of MDD and the effects of 8 weeks successful antidepressant treatment, suggesting these abnormalities may play an important role in the neuropathophysiology of MDD at its onset.

## Introduction

Convergent evidence from functional magnetic resonance imaging (fMRI) [1], [2], diffusion tensor imaging (DTI) [3] and positron emission tomography (PET) [4] studies suggest that dysregulation of frontal-subcortical circuits which involved with emotional and cognitive processing [5], [6] may contribute to the pathophysiology of major depressive disorder (MDD) (see review [7] and [8]). Gray matter volume (GMV) deficits have also been reported in MDD patients using structural magnetic resonance imaging (sMRI), including reductions in the dorsolateral prefrontal cortex (DLPFC) [9], [10], orbitofrontal cortex (OFC) [11], anterior cingulate cortex (ACC) [12], [13], [14], hippocampus [15], amygdala [14], [16], insula [17] and thalamus [10], [18], [19]. However, inconsistent findings with increased GMV in ACC [20] and thalamus [21] in MDD indicate that heterogeneity in sample age, medication exposures, age of onset, illness duration and number of acute episodes as well as different methodologies such as region of interest (ROI) and voxel-based morphometry (VBM) may contribute to the differences in results.

Antidepressants such as selective serotonin reuptake inhibitors (SSRIs), including fluoxetine, sertraline, paroxetine, citalopram and fluvoxamine, have been widely used for the treatment of MDD and have been reported to reverse functional abnormalities of frontal-subcortical circuits [22], [23], [24]. These findings confirm the key role of frontal-subcortical circuits in the pathophysiology of MDD and further support the hypothesis that specific treatment effects to these circuits may be one of the pharmacological mechanisms of antidepressants. Additionally, postmortem and sMRI studies reported that medicated MDD patients had more neural numbers [25], [26] or volumes [27] compared with unmedicated patients and HC, which suggest that antidepressants may have similar effects to related brain structure as well as its function. Until now, only few studies using ROI method directly evaluated the GMV changes by antidepressants. Frodl et al reported increased hippocampal volumes in MDD patients who took antidepressants over 3 years [28], however, no significant volume changes were observed in MDD patients after 10 months antidepressants treatment [29]. Therefore, further studies using VBM method to analyze whole brain GMV changes are necessary to better investigate the effects of antidepressants in MDD.

In the present study, we used VBM method to investigate whole brain structural abnormalities in single episode, medication-naïve MDD patients. Furthermore, we investigated the effects of an 8 weeks pharmacotherapy with fluoxetine, an antidepressant of SSRIs. We hypothesized that MDD patients would demonstrate GMV abnormalities in frontal-subcortical circuits such as DLPFC, OFC, ACC, insula, hippocampus, amygdala and thalamus, compared with HC. In addition, these abnormalities would be normalized after fluoxetine treatment.

## Methods

### Participants

28 MDD participants were recruited from outpatients at the Department of Psychiatry, First Affiliated Hospital of China Medical University. All MDD participants were diagnosed by two trained psychiatrists individually using the Structured Clinical Interview for DSM-IV [30] and met the following inclusion criteria: fulfilling DSM-IV criteria for major depressive disorder, single depressive episode; illness duration less than 3 months; aged 18 to 45; no comorbid other Axis I or II psychiatric disorders; currently experiencing an episode of depression with the score of at least 17 on the 17-item Hamilton Depression Rating Scale (HDRS-17) [31]; and no history of psychotropic medication, electroconvulsive therapy or psychotherapy.

28 HC matched for sex, age and education were recruited through the advertisements. The Structured Clinical Interview for DSM-IV confirmed the absence of DSM-IV Axis I or II psychiatric disorders. HC with a history of mood disorders in their first-degree family members were excluded. Exclusion criteria for all participants included: less than 18 years of age; any MRI contraindications; history of head injury or neurological disorder; any concomitant medical disorder. All participants were right-handed, underwent baseline clinical assessment and MRI scan within 48 hours of initial contact. All participants provided written informed consent after detailed description of the study. The study was approved by the Ethics Committee of China Medical University.

### Antidepressant Treatment and follow-up study

After the baseline MRI scan, MDD participants received 8 weeks outpatient fluoxetine treatment and outpatient interviews for every 2 weeks. Dosage of fluoxetine started with 10 mg/d, added to 20 mg/d after 1 week. After the 4 weeks visit, the dosage remained 20 mg/d or added maximum to 40 mg/d depending on the patient’s response and tolerance for the next 4 weeks. MDD participants did not take anxiolytic, antipsychotic, psychotherapy or other comedications during 8 weeks antidepressant treatment. Follow-up clinical assessment and MRI scan were completed for the MDD participants after 8 weeks antidepressant treatment.

### MRI acquisition and processing

All MRI scans were performed on a GE Signa 1.5 T MR scanner (General Electric, Milwaukee, USA) at the First Affiliated Hospital of China Medical University, Shenyang, China. Head motion was minimized with restraining foam pads. A standard head coil was used for radiofrequency transmission and reception of the nuclear magnetic resonance signal. Axial T1-weighted Fast Spoiled Gradient Echo (FSPGR) images were acquired with the following parameters: TR = 8.8 ms; TE = 4.2 ms; matrix = 512×512; FOV = 256×256 mm^2^; flip angle = 15°C; slice thickness = 1.0 mm without gap; 164 slices.

Images were processed and analyzed using the Diffeomorphic Anatomical Registration Through Exponentiated Lie algebra (DARTEL) [32], an improved VBM method which can achieve inter-subject brain images registration more accurately in Statistical and Parametric Mapping 8 (SPM8) (http://www.fil.ion.ucl.ac.uk/spm): (1) MR images were segmented into GM, white matter (WM) and cerebrospinal fluid using the standard unified segmentation model in SPM8; (2) The study-specific GM templates were created from the entire image dataset using DARTEL technique; (3) after an initial affine registration of the GM DARTEL templates to the tissue probability maps in Montreal Neurological Institute (MNI) space, non-linear warping of GM images was performed to the DARTEL GM template in MNI space and then employed in the modulation step to ensure that relative volumes of GM were preserved following the spatial normalization procedure; (4) the modulated, normalized GM images (representing GMV, voxel size 1.5×1.5×1.5 mm^3^) were smoothed with an 8-mm full width at half maximum isotropic Gaussian kernel [33].

### Statistical analysis

One-way ANOVA and X^2^ tests were used to compare demographic data and HDRS scores among the medication-naïve MDD, treated MDD and HC groups with SPSS 13.0 software (SPSS Inc, Chicago, Illinois). One-way ANOVA analysis was performed to investigate the differences of GMV among three groups using the framework of the General Linear Model (GLM) in a voxel-by-voxel manner with SPM8. An absolute threshold mask of 0.2 was employed in model specification. If the effects of group were significant, post-doc analyses of two sample t-tests were performed in SPM8, separately for every 2 groups (medication-naïve MDD *VS* HC, treated MDD *VS* HC, medication-naïve MDD *VS* treated MDD). Furthermore, for MDD patients who completed 8 weeks fluoxetine treatment and finished the follow-up MRI scan, a exploratory paired t-test was performed to investigate longitudinal differences. The findings were considered significant at a height threshold of P<0.05, corrected with false discovery rate (FDR) [34] for multiple comparisons and an extension threshold of 50 voxels (168 mm^3^). The mean volumes of the clusters shown differences in MDD groups were extracted using MarsBaR toolbox (http://marsbar.sourceforge.net/). Bivariate Pearson correlation analyses were performed in MDD groups to assess the correlation of HDRS scores and illness duration with mean volumes of the clusters.

## Results

28 MDD patients and 28 HC finished the baseline MRI scan. During the follow-up, 2 patients refused the antidepressant treatment after the baseline MRI scan and chose to receive other therapies, 2 patients refused follow-up MRI scan after treatment. 24 patients completed 8 weeks fluoxetine treatment (mean dosage 31.43±10.07 mg/d, range 10–40 mg/d) and finished the follow-up clinical assessment and MRI scan. There were no significant differences in age [F (2, 77)  = 2.92, p = 0.06], gender [F (2, 77)  = 1.50, p = 0.23], or education [F (2, 77)  = 0.54, p = 0.58] between 3 groups. The MDD group had the significant higher HDRS scores than the HC group which decreased significant after treatment [F (2, 77)  = 560, p = 0.000] (Table 1).

**Table 1:** Demographic and clinical data of subjects.

	Healthy Controls (n = 28)	Medication-naïve MDD Participants (n = 28)	Treated MDD participants (n = 24)	
Gender (male/female)	14/14	11/17	10/14	F (2, 77) = 1.50, p = 0.23
Age (years, mean ± S.D.) [range]	32.07±9.27 [18]–[44]	34.42±8.24 [18]–[43]	36.12±5.73 [24]–[43]	F (2, 77) = 2.92, p = 0.06
Education (years, mean ± S.D.) [range]	12.36±3.16 [6]–[16]	11.79±3.47 [6]–[16]	12.75±3.49 [6]–[16]	F (2, 77) = 0.54, p = 0.58
HDRS (mean ± S.D.) [range]	0.57±0.63 [0–2]	21.64±3.52 [17]–[26]	3.42±2.55 [0–8]	F (2, 77) = 560, p = 0.000
Illness duration (Month, mean ± S.D.) [range]	N/A	2.11±0.9 [1]–[3]	4.12±0.89 [3]–[5]	

S.D.: standard deviation.

MDD: major depressive disorder.

HDRS: Hamilton Depression Rating Scale.

**Figure 1:**
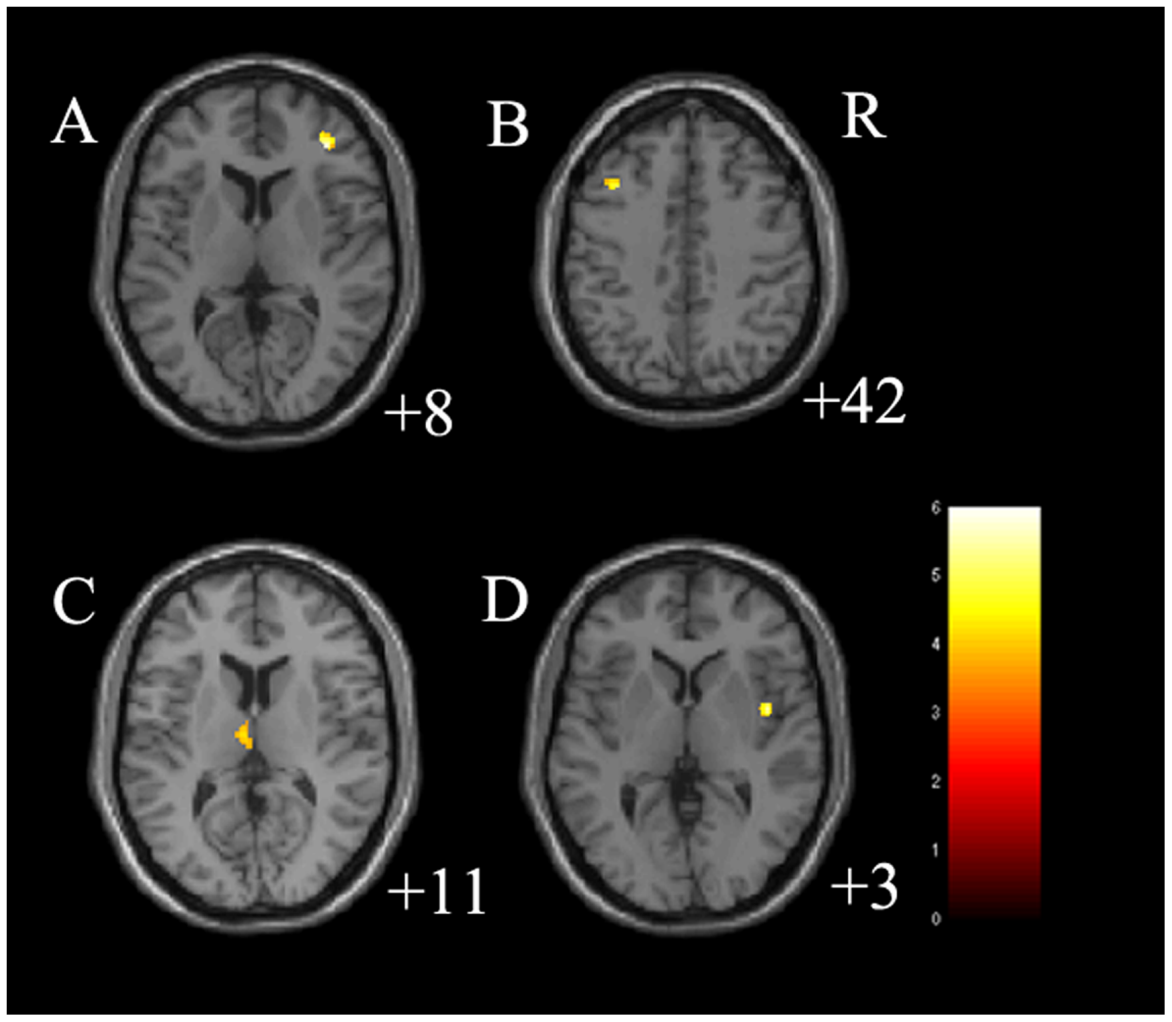
Regions of differences in gray matter volume in medication-naïve participants with major depressive disorder. A: the axial image (z = 8 mm Montreal Neurological Institute coordinate plane) shows the regions of significantly decreased gray matter volume in right dorsolateral prefrontal cortex in medication-naïve participants with major depressive disorder, compared to healthy controls (p<0.05, corrected). B: the axial image (z = 42 mm Montreal Neurological Institute coordinate plane) shows the regions of significantly decreased gray matter volume in left middle frontal gyrus in medication-naïve participants with major depressive disorder, compared to healthy controls (p<0.05, corrected). C: the axial image (z = 11 mm Montreal Neurological Institute coordinate plane) shows the regions of significantly increased gray matter volume in left thalamus in medication-naïve participants with major depressive disorder, compared to healthy controls (p<0.05, corrected). D: the axial image (z = 3 mm Montreal Neurological Institute coordinate plane) shows the regions of significantly increased gray matter volume in right insula in medication-naïve participants with major depressive disorder, compared to healthy controls (p<0.05, corrected). The color bar represents the range of *T* values. R =  right.

**Figure 2:**
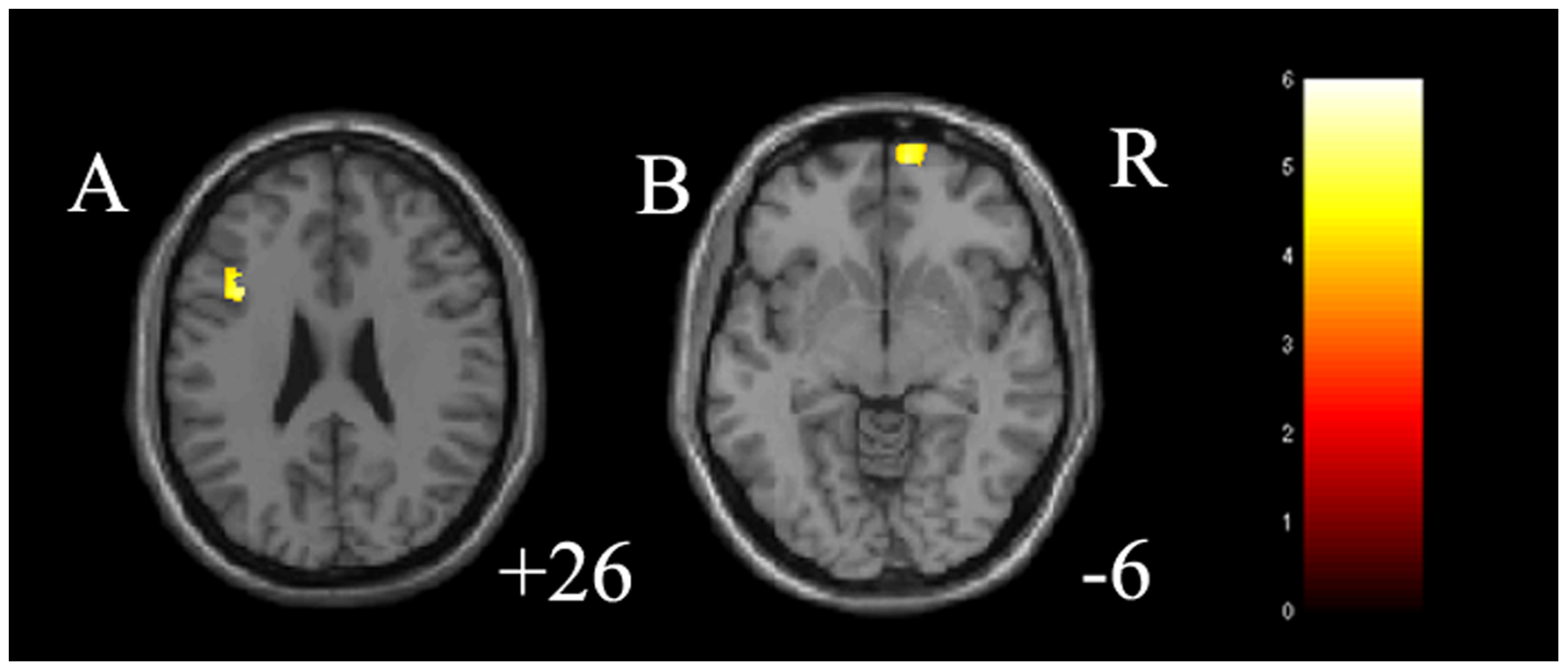
Regions of differences in gray matter volume in treated participants with major depressive disorder. A: the axial image (z = 26 mm Montreal Neurological Institute coordinate plane) shows the regions of significantly increased gray matter volume in left thalamus in treated participants with major depressive disorder, compared to healthy controls (p<0.05, corrected). B: the axial image (z = −6 mm Montreal Neurological Institute coordinate plane) shows the regions of significantly increased gray matter volume in right orbitofrontal cortex in treated participants with major depressive disorder, compared to healthy controls (p<0.05, corrected). The color bar represents the range of *T* values. R =  right.

The main effect between 3 groups on GMV was significant in the right DLPFC (cluster size  = 68 voxels, maximal point MNI coordinate: x = 37 mm, y = 40 mm, z = 8 mm, F (2, 77)  = 16.64, p<0.05, corrected), left middle frontal gyrus (cluster size  = 137 voxels, maximal point MNI coordinate: x = −36 mm, y = 15 mm, z = 26 mm, F (2, 77)  = 15.45, p<0.05, corrected) and right insula (cluster size  = 50 voxels, maximal point MNI coordinate: x = 43 mm, y = 0 mm, z = 3 mm, F (2, 77)  = 15.14, p<0.05, corrected) (Table 2). Post-hoc two-sample t-tests indicated medication-naïve MDD had significantly decreased GMV in the right DLPFC (cluster size  = 112 voxels, maximal point MNI coordinate: x = 37 mm, y = 40 mm, z = 8 mm, T (1, 77)  = 5.64, p<0.05, corrected), left middle frontal gyrus (cluster size  = 82 voxels, maximal point MNI coordinate: x = −36 mm, y = 19 mm, z = 42 mm, T (1, 77)  = 4.69, p<0.05, corrected) as well as increased GMV in the left thalamus (cluster size  = 93 voxels, maximal point MNI coordinate: x = −5 mm, y = −14 mm, z = 11 mm, T (1, 77)  = 3.78, p<0.05, corrected) and right insula (cluster size  = 85 voxels, maximal point MNI coordinate: x = 43 mm, y = 0 mm, z = 3 mm, T (1, 77)  = 5.31, p<0.05, corrected) compared to HC (Table 2 and Figure 1). Moreover, post-hoc two-sample t-tests indicated treated MDD had significantly increased GMV in the left middle frontal gyrus (cluster size = 340 voxels, maximal point MNI coordinate: x = −36 mm, y = 15 mm, z = 26 mm, T (1, 77)  = 5.10, p<0.05, corrected) and right OFC (cluster size  = 372 voxels, maximal point MNI coordinate: x = 11 mm, y = 63 mm, z = −6 mm, T (1, 77)  = 4.85, p<0.05, corrected) compared to HC (Table 2 and Figure 2). No difference on GMV was detected between medication-naïve MDD group and treated MDD group. The paired t-test for MDD patients before and after treatment did not detect any difference on GMV. In further correlation analysis, no correlation was detected between clinical variables and GMV in MDD group (Table 3).

**Table 2:** Areas with significant differences on gray matter volume in single episode participants with major depressive disorder compared to health control participants.

		MNI coordinates			
Areas	Cluster Size	x	y	z	F/T values
**Between 3 groups**					
Right dorsolateral prefrontal cortex	68	37	40	8	16.64
Left middle frontal gyrus	137	−36	15	26	15.45
Right insula	50	43	0	3	15.14
**nMDD<HC**					
Right dorsolateral prefrontal cortex	112	37	40	8	5.64
Left middle frontal gyrus	82	−36	19	42	4.69
**nMDD>HC**					
Left thalamus	93	−5	−14	11	3.78
Right insula	85	43	0	3	5.31
**tMDD>HC**					
Left middle frontal gyrus	340	−36	15	26	5.10
Right orbitofrontal cortex	372	11	63	−6	4.85

nMDD: medication-naïve major depressive disorder.

tMDD: treated major depressive disorder.

HC: healthy controls.

**Table 3:** Results of correlation analysis between clinical variables and GMV in MDD group.

Areas	HDRS scores	Illness duration
**nMDD**		
Right dorsolateral prefrontal cortex	r = 0.231, p = 0.238	r = 0.025, p = 0.901
Left middle frontal gyrus	r = −0.108, p = 0.585	r = 0.208, p = 0.289
Left thalamus	r = 0.327, p = 0.089	r = −0.298, p = 0.123
Right insula	r = 0.258, p = 0.185	r = −0.222, p = 0.256
**tMDD**		
Left middle frontal gyrus	r = 0.093, p = 0.637	r = 0.051, p = 0.798
Right orbitofrontal cortex	r = 0.291, p = 0.132	r = −0.030, p = 0.880

GMV: gray matter volume.

HDRS: Hamilton Depression Rating Scale.

nMDD: medication-naïve major depressive disorder.

tMDD: treated major depressive disorder.

## Discussion

The main findings of this study were of the decreases of GMV in the right DLPFC and left middle frontal gyrus, as well as the increases of GMV in the left thalamus and right insula in single episode, medication-naïve MDD participants with illness duration less than 3 months compared with HC. Furthermore, an increase of GMV in the left middle frontal gyrus and right OFC was noted in MDD participants after 8 weeks fluoxetine treatment compared with HC. To our knowledge, this study provides the first evidence of structural abnormalities in frontal-subcortical circuits and short-term effects with antidepressant therapies in an MDD sample with minimal influences of chronicity or treatment related confounds.

The dorsolateral prefrontal circuit is one of frontal-subcortical circuits originating from prefrontal cortex, through striatum to thalamus then back to prefrontal cortex as well as connecting other functional related brain areas such as middle frontal area (Brodmann 8) and temporal area (including insula) [5]. Dysfunction of dorsolateral prefrontal circuit is involved in some depressive syndromes such as psychomotor retardation, impaired attention and executive dysfunction [35] and have been implicated in MDD in neuroimaging studies [23], [36], [37]. Several sMRI studies also demonstrated gray matter changes in these areas in MDD [9], [10], [38], [39]. Chang et al reported decreased GMV in bilateral DLPFC in late-life depression [40] and Amico et al reported decreased GMV in DLPFC in both MDD and healthy controls with a positive family history for MDD [41]. In our previous study using diffusion tensor imaging (DTI), we also found that MDD showed abnormalities of white matter fibers which comprise the interconnection within dorsolateral prefrontal circuit [42]. Taken together, our findings of decreased GMV in the right DLPFC and left middle frontal gyrus in MDD are consistent with these findings and further support that dysfunction of dorsolateral prefrontal circuit may play an important role in MDD pathophysiology.

Interestingly, in the current study, we found increased GMV in the left thalamus and right insula in single episode, medication-naïve MDD participants. Structural abnormalities about thalamus and insula in MDD are controversial in previous studies. Kim et al reported lower GMV in bilaterally thalamus in female participants with MDD [19] and Turner et al reported smaller mean right thalamus volume in MDD [18]. Another study with older depressive patients detected gray matter reductions in the insula which were associated with the number of relapses [17]. However, a recent study with first-episode, drug naïve MDD patients showed increased GMV in right thalamus consistent with our results [21]. Since the increased thalamic GMV found in our study and Zhang’s were not likely the results of differences in numbers of episode or medication exposure as the MDD participants were single episode, medication naïve in both studies, we speculate that the increased volume of thalamus and insula may be involved in the early stage of MDD and not likely to be the result of medication exposure. Another explanation is that increased GMV may also be related to preapoptotic osmotic changes or hypertrophy, marking areas of early neuronal pathology [20], [43]. In conclusion, our findings about GMV abnormalities in single episode, medication-naïve MDD participants suggest that structural abnormalities in frontal-subcortical circuits may be present in the early stages of MDD and play an important role in the development of MDD pathophysiology.

In the current study, we found that after 8 weeks antidepressant treatment, MDD participants detected increased GMV in the left middle frontal gyrus and right OFC compared with HC. Our findings about the effects of short-term antidepressant treatment are in accordance with another long time follow up study in which increased hippocampal volume was detected in MDD patients who took antidepressants over the 3 full years [28]. Additionally, recent fMRI studies showed that decreased DLPFC activation [22] and increased thalamus activation [23] during emotion processing in MDD could be normalized after 8 weeks antidepressant treatment. As the neurobiological hypothesis that antidepressants may take its effects through enhancing neuroplasticity and neurogenesis in specific brain regions [44], [45] especially frontal cortex [46], we speculate that medication-induced changes are likely to be observed in structure as in function, which may be detected after short time treatment in MDD. However, the confound findings such as Vythilingam et al reported no significant differences in hippocampal volume after 7±3 months antidepressant treatment [29] in MDD as well as Janssen et al reported no cerebral volume differences after 12 weeks treatment with venlafaxine or nortriptyline in late life depression [47] suggest that medication-induced changes in MDD should be investigated with long time longitudinal imaging studies in future.

In this study, we did not find GMV abnormalities in ACC, amygdala or hippocampus in MDD, which are consistently reported in previous studies in MDD [48]. Our explanation is that these findings are more correlated with multiple depressive episodes, medication exposures and illness duration [49], [50], [51]. Our previous findings in a chronic sample [14], as well as findings by Zou et al [15] suggest that illness duration may be a key influential factor in gray matter abnormalities in MDD. Future studies including individuals with different illness durations are helpful to investigate this hypothesis.

This study had some limitations that should be noted. First is the relative small sample size and short time follow-up design, which may be related to the disability to find the relationship between clinical variables and neuroimaging results. Secondly, since all 24 patients who finished the second scan showed significant response to antidepressants, there is the potential for selection bias, in that only participants with good clinical outcomes chose to remain involved in this study, thereby limiting the generalization of our results. Future studies with large sample size and longtime follow-up design to compare the differences between responders and non-responders in MDD is necessary. Furthermore, one must be cautious to our discussion about medication-induced changes in MDD because we simply reported increased GMV in MDD participants after 8 weeks fluoxetine treatment compared with HC, but no differences compared with treat-naïve MDD at baseline detected. It is critical to directly compare GMV between before and after treatment to further examine our findings using paired test in MDD. Finally, the technical limitation of VBM method which exhibit lower accuracy during segmentation of some subcortical structures such as thalamus suggests our results need to be further investigated with complementary sMRI techniques such as cortical thickness and tensor-based morphometry in future [52], [53].

In summary, our study of single episode, medication-naïve MDD subjects demonstrated structural abnormalities of frontal-subcortical circuits in the early stage of MDD and the effects of 8 weeks successful antidepressant treatment. Future studies with treatment-naïve and treated MDD, or remitted and active MDD combining other techniques such as DTI and fMRI could further elucidate the role of frontal-subcortical circuits abnormalities in the neuropathophysiology of MDD.
